# Clinical and genetic characteristics of three patients with congenital insensitivity to pain with anhidrosis: Case reports and a review of the literature

**DOI:** 10.1002/mgg3.2430

**Published:** 2024-04-05

**Authors:** Jun Hee Cho, Soojin Hwang, Yoon Hae Kwak, Mi‐Sun Yum, Go Hun Seo, June‐Young Koh, Young Seok Ju, Ji‐Hee Yoon, Minji Kang, Hyo‐Sang Do, Soyoung Kim, Gu‐Hwan Kim, Hyunwoo Bae, Beom Hee Lee

**Affiliations:** ^1^ Medical Genetics Center, Asan Medical Center University of Ulsan College of Medicine Seoul Republic of Korea; ^2^ Department of Pediatrics, Asan Medical Center Children's Hospital University of Ulsan College of Medicine Seoul Republic of Korea; ^3^ Department of Orthopedic Surgery, Asan Medical Center Children's Hospital University of Ulsan College of Medicine Seoul Republic of Korea; ^4^ Department of Pediatric Neurology Asan Medical Center, University of Ulsan College of Medicine Seoul Republic of Korea; ^5^ Division of Medical Genetics, 3billion, Inc. Seoul Republic of Korea; ^6^ Genome Insight, Inc. San Diego California USA; ^7^ Asan Medical Center Asan Institute for Life Sciences Seoul Republic of Korea

**Keywords:** congenital insensitivity to pain with anhidrosis, genotype–phenotype correlation, hereditary sensory and autonomic neuropathy, *NTRK1* gene

## Abstract

**Background:**

Congenital insensitivity to pain with anhidrosis (CIPA) is an extremely rare autosomal recessive disorder caused by loss‐of‐function mutations of the *NTRK1* gene, affecting the autonomic and sensory nervous system. Clinical manifestation is varied and includes recurrent fever, pain insensitivity, anhidrosis, self‐mutilating behavior, and intellectual disability.

**Methods:**

Clinical and genetic features were assessed in two males and one female with genetically confirmed CIPA using exome or genome sequencing.

**Results:**

CIPA symptoms including recurrent fever, pain insensitivity, and anhidrosis manifested at the age of 1 year (age range: 0.3–8 years). Two patients exhibited self‐mutilation tendencies, intellectual disability, and developmental delay. Four *NTRK1* (NM_002529.3) mutations, c.851‐33T>A (p.?), c.2020G>T (p.Asp674Tyr), c.2303C>T (p.Pro768Leu), and c.574‐156_850+1113del (exons 5‐7 del) were identified. Two patients exhibited early onset and severe phenotype, being homozygous for c.851‐33T>A (p.?) mutations and compound heterozygous for c.851‐33T>A (p.?) and c.2020G>T (p.Asp674Tyr) mutation of *NTRK1*. The third patient with compound heterozygous mutations of c.2303C>T (p.Pro768Leu) and c.574‐156_850+1113del (exons 5‐7 del) displayed a late onset and milder clinical manifestation.

**Conclusion:**

All three patients exhibited variable phenotypes and disease severity. This research enriches our understanding of clinical and genetic aspects of CIPA, highlighting variable phenotypes and disease severity.

## Graphical abstract

Patients with congenital insensitivity to pain with anhidrosis typically present with cellulitis in their fingers and toes, often leading to amputation due to self‐mutilating behavior. Osteodystrophy can result from a history of recurrent infections and surgeries.

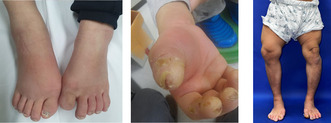

## 1 INTRODUCTION

Congenital insensitivity to pain with anhidrosis (CIPA, MIM #256800) is a rare autosomal recessive genetic disorder initially reported by Swanson (1963) in two male siblings who lacked pain sensitivity and did not sweat (Swanson, 1963). Key clinical features include impaired pain perception, anhidrosis, recurrent febrile episodes, and intellectual disability (Indo, 1993). Impaired pain perception leads to recurrent injuries, burns, and self‐mutilating behaviors such as biting the tongue, fingers, and toes. Repeated trauma or self‐mutilating behaviors increase the risk of fracture, arthropathy, and musculoskeletal infection (Echaniz‐Laguna et al., 2021; Li et al., 2019). Anhidrosis disturbs thermoregulation in hot environments, resulting in recurrent febrile episodes. Most CIPA patients exhibit psychiatric disabilities, including intellectual disability and attention deficit hyperactivity disorder (ADHD; Indo, 2018).

CIPA, also known as hereditary sensory and autonomic neuropathy type 4 (HSAN type 4), is caused by mutations in the *NTRK1* gene on chromosome 1q23.1, spanning over 25 kb with 17 exons and 16 introns. It encodes Tropomyosin‐related kinase A (TrkA) protein, a membrane‐bound receptor tyrosine kinase with a high affinity for nerve growth factor (NGF) crucial for nociceptive reception and sweat regulation (Indo et al., 1996; Indo, 2001). NGF bindings to TrkA activate signaling pathways vital for the survival and maintenance of peripheral sensory and autonomic neurons during embryonic development (Glebova & Ginty, 2005; Kristiansen & Ham, 2014). Ad‐ and C‐fibers and sympathetic postganglionic neurons are dependent on NGF‐TrkA signaling. As primary afferent fibers of small diameters (Ad and C) transmit pain sensation and sympathetic postganglionic neurons innervate eccrine glands, CIPA patients exhibit deficits in pain sensation and sweating (Indo, 2012).

Over 115 pathogenic or likely pathogenic *NTRK1* variations have been reported (http://www.hgmd.cf.ac.uk/ac/), including nonsense, frameshift, splice‐site, missense, and gross deletion variants in the extracellular and intracellular domains (Indo, 2001). Genotype–phenotype correlation studies show heterogeneity, while some groups report no correlation (Indo, 2002; Mardy et al., 1999), some groups identify association based on molecular structure and function of the *NTRK1* gene. Patients with homozygous p.Met581Val variants, for instance, may exhibit normal body temperature and attenuated phenotypes (Miranda, Selleri, et al., 2002; Wang et al., 2018; Yotsumoto et al., 1999).

This study describes the clinical manifestations of CIPA in three Korean patients from unrelated families. Through exome sequencing or genome sequencing, four *NTRK1* variants, c.851‐33T>A (p.?), c.2020G>T (p.Asp674Tyr), c.2303C>T (p.Pro768Leu), and c.574‐156_850+1113del (exons 5‐7 del), were identified. The patients exhibited variable phenotypes and disease severity. This study widens the clinical and genetic spectrum, enhancing our understanding of this rare condition.

## 2 METHODS

### 2.1 Patients and clinical assessment

A total of three Korean patients from three unrelated nonconsanguineous families were diagnosed with CIPA at Asan Medical Center, Seoul, Republic of Korea from September 2015 to August 2023. Medical histories, physical examination findings, developmental assessments, blood tests, and image studies were reviewed based on electronic medical records. A pedigree of each family was obtained based on clinical assessment and genetic test results.

### 2.2 Genetic analysis

Peripheral blood samples were obtained from each proband and their parents. Genomic DNA was extracted from whole blood using Allprep DNA/RNA kits (Qiagen, Venlo, Netherlands). Exome sequencing (ES) and genome sequencing (GS) were performed for families #1 and #2 and family #3, respectively. For ES, the exon regions of genomic DNA were targeted using the xGen Exome Research Panel v2 (Integrated NDA Technologies, Coralville, Iowa, USA). DNA libraries were prepared using the TruSeq DNA PCR‐Free Library Prep Kits (Illumina, San Diego, CA, USA) for GS. Sequencing was performed on the Illumina NovaSeq 6000 System (San Diego, CA, USA) as 150 bp paired‐end reads. Data from ES and GS were aligned to the GRCh38 and GRCh37 human reference genomes, respectively, using the BWA‐MEM algorithm. Single nucleotide variants (SNVs) and small insertion/deletion (INDEL) variants were called using GATK v.3 and Strelka2. Structural variants were detected using Delly. Variants were then annotated by Ensembl Variant Effect Predictor (VEP) and filtered and classified by the RareVision system (Genome Insight, San Diego, CA, USA) for GS and EVIDENCE (3billion Inc., Seoul, South Korea) for ES following the American College of Medical Genetics and Genomics (ACMG) and the Association for Molecular Pathology (AMP) guidelines (Richards et al., 2015; Seo et al., 2020; Yun et al., 2023). The filtered and classified variant list was manually reviewed by medical geneticists and physicians. The most likely variants that can explain the patient's phenotype and family history were selected for reporting. Sanger sequencing was performed for the family members on the variant identified by ES and GS. It was performed using primers designed to appropriate exons and BigDye Terminatore V3.1 Cycle Sequencing kit (Applied Biosystems, Foster City, CA, USA) according to the manufacturer's instructions. Electrophoresis and analysis of the reaction mixtures were done with ABI 3500xL Genetic analyzer (Applied Biosystems).

### 2.3 Literature review

The literature review was conducted using the following steps: The biomedical and life science database (PubMed) was searched using the MeSH Terms (congenital insensitivity to pain with anhidrosis [MeSH Terms]) OR (Hereditary sensory autonomic neuropathy type 4 [MeSH Terms]). All English articles between November 1951 and August 2023 were assessed. Articles reporting three or more of the symptoms of pain insensitivity, anhidrosis, recurrent fever, and intellectual disability in genetically diagnosed patients with CIPA were included. Symptoms not described were considered absent for the patient. When genetic analysis was not conducted for all reported patients, only results from genetically diagnosed patients were included. Articles reporting surgical and anesthetic procedures for previously diagnosed CIPA patients were excluded.

## 3 RESULTS

### 3.1 Clinical presentation of the three patients

**FIGURE 1:**
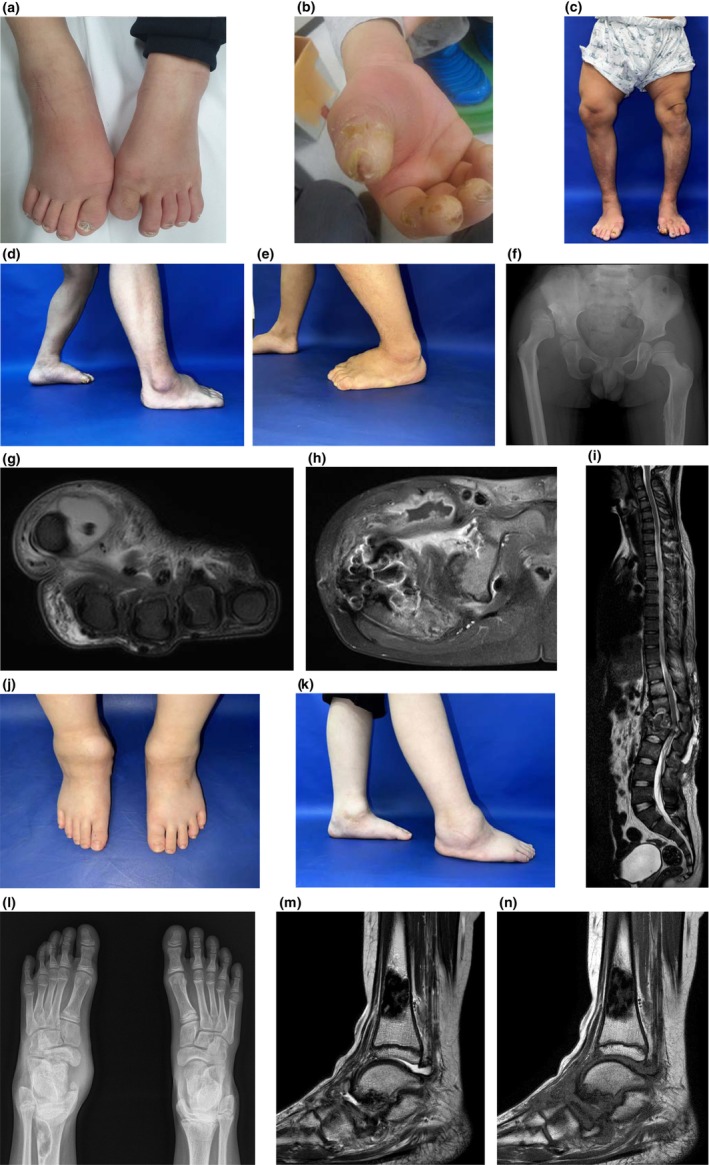
Clinical features of congenital insensitivity to pain with anhidrosis patients. (a–i) Patient 1: (a, b) finger and toe cellulitis and amputation due to self‐mutilating behavior, (c–e) recurrent infection and operation history led to osteodystrophy of lower extremities and disproportionate short stature, (f) right hip joint dislocation and osteodystrophy, (g) tenosynovitis of right flexor pollicis longus tendon, (h) right inguinal abscess, (i) paravertebral abscess, (T11‐L5) (j–n) Patient 3: (j, k) right foot swelling due to osteodestruction, (l) early destruction of both talonavicular joints, (m, n) left distal tibia nonossifying fibroma.

The median age at presentation was 12 months (range, 4 months–8 years). The median age at diagnosis was 8 years (range, 9 months–16 years). The clinical features of each patient are described in Table 1 and Figure 1.

**TABLE 1:** Clinical and genetic features of the three patients with CIPA.

	Patient 1	Patient 2	Patient 3
Gestational age	38 weeks and 2 days, 2750 gm	40 weeks, 2850 gm	40 weeks, 3560 gm
Age at first visit	12 months	4 months	8 years
Age at diagnosis	2 years (clinical), 16 years (genetic)	9 months	8 years
Gender	Male	Male	Female
Insensitivity to pain	(+) Lower extremities	(+) From birth	(+) but partially preserved
Anhidrosis or recurrent fever	(+) Lower extremities	(+) From birth	(+) no sweating except back
Developmental delay or intellectual disability	Global development delayMild mental retardation	Global development delay	Normal development Normal intelligence Aggressive behavior
Bone fracture orjoint deformity	Developmental dysplasia of the hip, Right hip dislocation Osteodystrophy Paraplegia Disproportionate short stature		Left distal tibia fracture Left talonavicular joint deformity Left tibia nonossifying fibroma
Self‐mutilation	(+) nail biting	(+) nail biting	
Infection	Paravertebral abscess, osteomyelitis		
Mutation 1	c.851‐33T>A (p.?)	c.851‐33T>A (p.?)	c.2303C>T (p.Pro786Leu)
Mutation 2	c.851‐33T>A (p.?)	c.2020G>T (p.Asp674Tyr)	c.574‐156_850+1113del (exons 5‐7del)

Patient 1 was a single child born to healthy, nonconsanguineous Korean parents. He was born at a gestational age of 38 weeks, weighing 2750 g (5–10th percentile). His parents observed that his basal body temperature had been slightly high since birth, and he experienced recurrent fever episodes. He did not sweat at all until childhood. However, the symptoms were alleviated with the progression of age. At the age of 18 months, he abruptly showed a limping gait and was diagnosed with developmental dysplasia of the hip. At the age of 2 years, he jumped from the table, and his right hip was dislocated. However, he did not show any expected normal pain responses and actively moved his legs. His height and body weight were under the 3rd percentile throughout the growth period. At the age of 3 years, his developmental age was 24 months for cognition, 16 months for language expression, 24 months for language comprehension, 24 months for social, and 11 months for motor development. Although he started language and cognitive rehabilitation, he was diagnosed with mild mental retardation at the age of 6, and his grades in school were very low. He also started to bite his fingers and toes, and repetitive hospitalization was required due to recurrent soft tissue and bone infections, such as thigh abscess, hand or knee cellulitis, and foot osteomyelitis. At the age of 16 years, infectious spondylitis with paravertebral abscess was noted, with compressed T11‐L5 spines. Despite the immediate decompression surgery, he became paraplegic and was wheelchair‐bound. The length of his upper extremities was within the normal range, whereas his lower extremities were relatively short.

Patient 2 was born at the gestational age of 40 weeks to healthy, nonconsanguineous Korean parents weighing 2850 g (10–15th percentile). He was the only child in this family. At birth, a high‐arched palate and hypotonia were noted. Recurrent episodes of fever with a decrease in sweating were noted without any other symptoms, such as cough or rhinorrhea. He showed self‐mutilating behavior by tongue biting. No pain response was noted during the venipuncture or muscular injection. At the age of 7 months, his developmental age was 3 months for fine motor and 5 months for personal‐social areas. Although he started rehabilitation therapy, he was diagnosed with global developmental delay at 11 months of age. His developmental age was 8 months for gross motor, 6 months for fine motor, 8 months for personal social, 9 months for language, and 8 months for cognitive‐adaptive area. At the age of 4 years, his height was 103 cm (25th percentile), and his weight was 14.4 kg (3rd percentile). Anhidrosis, mild fever, self‐mutilation of the tongue, and poor weight gain persisted.

Patient 3 was born at a gestational age of 40 weeks to healthy, nonconsanguineous Korean parents weighing 3560 g (50–75th percentile). Her two older sisters were healthy. Recurrent episodes of fever with a decrease in sweating were noted, with no definite evidence of infection. At the age of 7 years, she wrenched her left ankle, but she did not feel the pain or experience limitations of motion. She went to the hospital 1 week after the accident because of sustained left foot swelling and was diagnosed with left distal femur fracture and left foot Kohler's disease. At 8 years old, a new hypolucent fibro‐osseous lesion in the left distal femur, destruction of both talonavicular joints, and avascular necrosis of the left navicular bone were observed. She subsequently underwent several surgeries due to the persistent nonunion of destructive bones. At the age of 10 years, her height was in the 85th percentile, and her weight was in the 90th percentile. Her intelligence was normal without any self‐mutilating behaviors, but her parents observed impulsive and aggressive tendencies.

### 3.2 Literature review

Among the 2114 searched articles, 47 were included, and the clinical features of 178 patients were reviewed. The results of our study were consistent with those of previous studies. The clinical features of these patients are presented in Table 2. The most prevalent symptom among genetically diagnosed CIPA patients was anhidrosis (97%), followed by insensitivity to pain (94%), and recurrent fever (89%). Intellectual disability or self‐mutilation was observed in 75% of patients, and musculoskeletal problems such as bone fracture or joint deformity were recorded in 64% of patients. Recurrent or severe infection, such as osteomyelitis, was reported in 24% of patients.

**TABLE 2:** Clinical features of patients in this study and those of previously reported patients with genetically diagnosed congenital insensitivity to pain with anhidrosis.

	Our study	Literature	Total
Patients	3	178	181
Male	2/3 (66)	118/178 (66)	120/181 (66)
Female	1/3 (33)	60/178 (34)	61/181 (34)
Insensitivity to pain	3/3 (100)	168/178 (94)	171/181 (94)
Anhidrosis	3/3 (100)	172/178 (97)	175/181 (97)
Recurrent fever	3/3 (100)	158/178 (89)	161/181 (89)
Intellectual disability	2/3 (66)	133/178 (75)	135/181 (75)
Bone fracture Joint deformity	2/3 (66)	113/178 (63)	115/181 (64)
Self‐mutilation	2/3 (66)	133/178 (75)	135/181 (75)
Recurrent Infection	1/3 (33)	42/178 (24)	43/181 (24)

### 3.3 Genetic analysis

**FIGURE 2:**
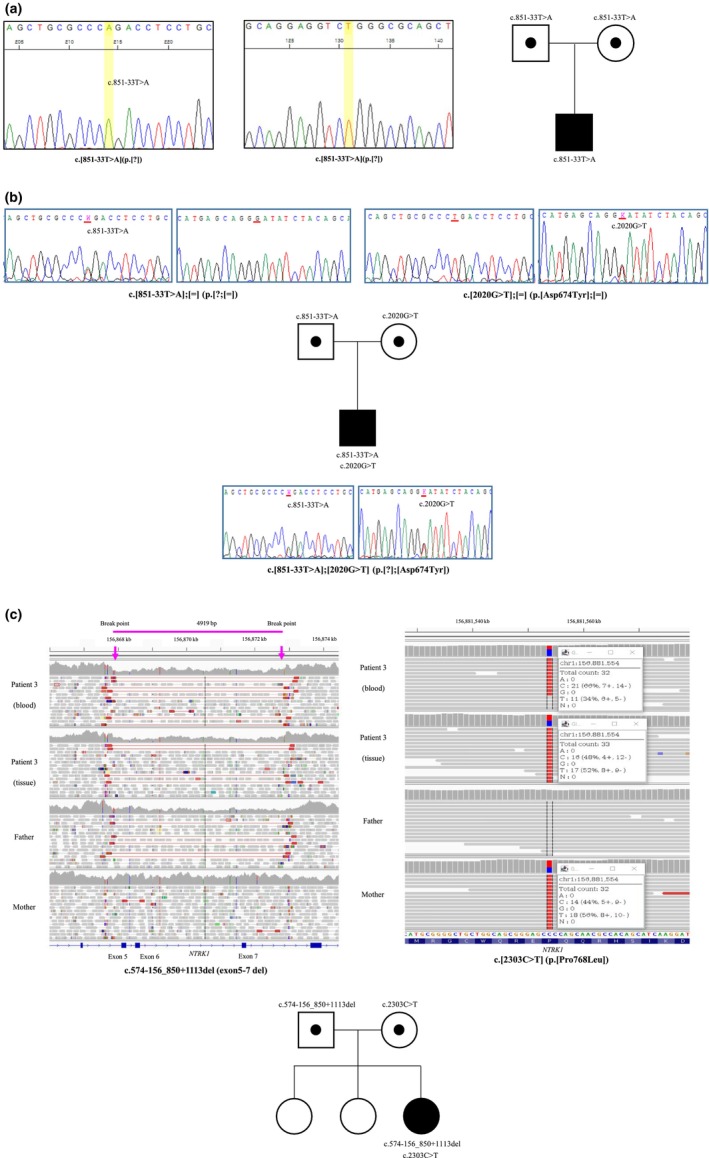
Genetic analysis and the pedigrees of congenital insensitivity to pain with anhidrosis (CIPA) patients. The samples of both parents of each patient were sequenced, and all parents were identified as asymptomatic carriers. Patient 1 and patient 2 did not have siblings, whereas patient 3 had two older sisters. The parents of patient 3 denied the presence of clinical features of CIPA in two older sisters, and sequencing was not performed. (a) Partial sequence of *NTRK1* of patient 1, who has homozygous mutation, c.[851‐33T>A];[851‐33T>A]. (b) Partial sequence of *NTRK1* of patient 2 and his parents. Patient 2 had c.[851‐33T>A];[2020G>T] (p.[?];[Asp674Tyr]) compound heterozygous variants. His father was a carrier of heterozygous c.[851‐33T>A];[=] variant and mother was a carrier of heterozygous c.[2020G>T];[=] (p.[Asp674Tyr];[=]) variant, respectively. (c) Genome sequencing of patient 3 who had c.[574‐156_850+1113del];[2303C>T] (p.[?];[Pro768Leu]) compound heterozygous variants and her carrier parents. Her father was a carrier of heterozygous c.[574‐156_850+1113del];[=] variant and mother was a carrier of heterozygous c.[2303C>T];[=] (p.[Pro768Leu];[=]) variant, respectively. Peripheral blood samples and bone tissues collected via biopsy were analyzed.

Exome sequencing was performed for patients 1 and 2 and their parents, and genome sequencing was performed for patient 3 and her parents. Figure 2 shows the pedigrees of three families and identified variants. Patient 1 was homozygous for c.851‐33T>A (p.?) in the *NTRK1* gene, and both of his parents were carriers for c.851‐33T>A (p.?). This variant was previously reported as pathogenic according to the ACMG classification, which led to aberrant splicing (Wang et al., 2018). Patient 2 showed compound heterozygous pathogenic variants, c.851‐33T>A (p.?) and c.2020G>T (p.Asp674Tyr). c.851‐33T>A (p.?) was inherited from his father, and the missense variant, c.2020G>T (p.Asp674Tyr), classified as likely pathogenic by ACMG classification, was inherited from his mother. c.2020G>T (p.Asp674Tyr) has also been reported previously (López‐Cortés et al., 2020). In patient 3, compound heterozygous variants c.2303C>T (p.Pro768Leu) and c.574‐156_850+1113del (exons 5‐7 del) were identified. c.2303C>T (p.Pro768Leu) was inherited from his mother, and gross deletion of c.574‐156_850+1113del (exons 5‐7 del) was inherited from her father. c.2303C>T (p.Pro768Leu) has been reported previously (Jung et al., 2013), while c.574‐156_850+1113del (exons 5‐7 del) has not been reported (Table 3).

**TABLE 3:** Genetic variants identified in three congenital insensitivity to pain with anhidrosis families. A total of four different variants were identified: 2 missense variants, 1 splice site, and 1 gross deletion. All three patients inherited two variants, one from each parent. Patient 1 and patient 2 had two variants that were located in the tyrosine kinase domain of *NTRK1.* Patient 3 had one variant in the tyrosine kinase domain (c.2303C>T) and one variant in exons 5–7 (c.574‐156_850+1113del). All variants identified were classified as pathogenic or likely pathogenic according to ACMG guidelines.

	Nucleotide change	Amino acid change	Zygosity	Exon/intron	ACMG classification	Inheritance
Patient 1	c.851‐33T>A		Homozygote	Intron 7	Pathogenic	Both parents
Patient 2	c.851‐33T>A		Heterozygote	Intron 7	Pathogenic	Paternal
	c.2020G>T	p.Asp674Tyr	Heterozygote	Exon 15	Likely pathogenic	Maternal
Patient 3	c.2303C>T	p.Pro768Leu	Heterozygote	Exon 17	Pathogenic	Maternal
	c.574‐156_850+1113del^*^		Heterozygote	Exons 5‐7 del	Likely pathogenic	Paternal

^*^Novel variant

## 4 DISCUSSION

CIPA is caused by mutations in the *NTRK1* gene, resulting in a lack of pain perception and anhidrosis. It belongs to hereditary sensory and autonomic neuropathy (HSAN), which encompasses a spectrum of inherited disorders characterized by sensory and autonomic dysfunction. HSAN subtypes are classified based on disease‐causing genes and sophisticated clinical phenotypes. CIPA is specifically designated as HSAN type 4, where *NTRK1* mutations lead to the loss of pain and temperature sensation, anhidrosis, recurrent fever, joint deformities, and intellectual disability (Yuan et al., 2013). However, diagnosing CIPA and differentiating it from other diseases, such as hypohidrotic ectodermal dysplasia or idiopathic generalized anhidrosis, which exhibit symptoms resembling anhidrosis, can be challenging in clinical practice (Sil et al., 2022). Genetic analysis is crucial for achieving accurate diagnoses and categorizing HSAN subtypes. Given the complexity of these phenotypes, it is essential to explore the genetic causes and understand the underlying molecular mechanisms.

*NTRK1* was initially discovered in 1986 as part of a novel tyrosine kinase family isolated from human colon carcinoma cells (Martin‐Zanca et al., 1986, 1989). *NTRK1* encodes the TrkA protein, which acts as a signal‐transducing receptor for NGF (Kaplan et al., 1991; Klein et al., 1991). The binding of NGF induces phosphorylation and homodimer formation of TrkA, leading to the activation of downstream intracellular signal transduction (Indo, 2001; Kaplan & Miller, 2000). NGF‐TrkA signaling plays crucial roles in the survival and growth of neurons, neuronal differentiation, neurite outgrowth, and synaptic plasticity (Reichardt, 2006). Primary afferent neurons and sympathetic postganglionic neurons in the peripheral nervous system, which form afferent and efferent pathways in thermal regulation, are governed by NGF (Indo, 2014, 2018). These NGF‐dependent neurons influence inflammation by secreting pro‐inflammatory or anti‐inflammatory cytokines (Indo, 2012, 2018; McMahon, 1996; Pezet & McMahon, 2006). Modulation of NGF is associated with sensitization of the nociceptor and altered transcription of the nociceptor genes, which could lead to hyperalgesia (Denk et al., 2017). In addition, NGF‐dependent neurons are considered to play a crucial role in emotions and feelings owing to their capacity to regulate the interoceptive system (Craig, 2002; Damasio & Carvalho, 2013; Indo, 2018).

TrkA exists as two isoforms of 790 or 796 amino acid residues. Except for the presence of six amino acid residues located near the extracellular transmembrane region, two isoforms have similar biological properties, including NGF binding (Barbacid, 1995). Yet, given that the isoform comprising 796 amino acid residues is abundantly expressed in neuromuscular tissues, whereas the other isoform is primarily expressed in nonneuronal cells, the former is deemed a more persistent isoform associated with the pathophysiology of CIPA (Barbacid, 1995; Schneider & Schweiger, 1991). The TrkA protein is composed of three parts: an extracellular domain, an intracellular domain, and a single transmembrane domain dividing them (Barbacid, 1995; Schneider & Schweiger, 1991). The extracellular domain comprises a signal peptide, two cysteine clusters, three tandem leucine‐rich motifs, and two immunoglobulin‐like domains crucial for NGF binding (Barbacid, 1995; Windisch et al., 1995). The intracellular domain plays a pivotal role in signal transduction following NGF binding and encompasses a juxtamembrane region, a tyrosine kinase domain, and a 15 amino acid carboxy‐terminal tail (Barbacid, 1995). Autophosphorylation sites identified at TrkA tyrosine residues 490, 670, 674, 675, and 785 serve as docking sites for downstream intracellular signal transduction molecules (Barbacid, 1995; Stephens et al., 1994).

Cardinal symptoms and signs of CIPA, including insensitivity to pain, anhidrosis, and mental retardation, result from the absence or deficiency of functional TrkA proteins (Indo, 2012). The binding of NGF activates TrkA tyrosine kinase activity, which results in neuronal growth and the prevention of neuronal apoptosis (Yuan & Yankner, 2000). NGF‐dependent primary afferent neurons with small diameters and thinly myelinated Ad‐fibers or unmyelinated C‐fibers transmit nociceptive and interoceptive stimuli, whereas sympathetic postganglionic neurons innervate various exocrine glands, including sweat glands (Indo, 2018). Patients with CIPA exhibited insensitivity to pain, anhidrosis, and recurrent fever as they lack NGF‐dependent primary afferents and sympathetic postganglionic neurons crucial for pain sensation and homeostasis maintenance (Indo, 2012, 2018). In the brain, TrkA is principally expressed in cholinergic neurons that are involved in learning and memory (Aboulkassim et al., 2011). TrkA density is relatively low in patients with mild cognitive impairment or Alzheimer's disease compared to that in the group without cognitive impairment (Mufson et al., 2000). Selective TrkA ligand treatment showed improvement in memory and learning in vivo (Bruno et al., 2004). NGF‐TrkA signaling is associated with the pathogenesis of Alzheimer's disease by modulating the metabolism of amyloid precursor protein and synaptic functions in cholinergic neurons (Canu et al., 2017). Additionally, the mRNA levels of TrkA in the brain were lower in individuals who died by suicide as a result of major depressive disorder, compared with those who died due to a traffic accident (Erbay et al., 2021). These findings indicate that TrkA‐expressing, NGF‐dependent neurons play a role in mediating diverse intellectual abilities. Dysfunction in NGF‐TrkA signaling may lead to mental retardation and psychiatric issues such as hyperactivity or emotional lability (Indo, 2012, 2018). This molecular mechanism contributes to the genetic etiology–phenotype association for HSAN. HSAN type V, caused by a mutation in the *NGF* gene, also exhibits a phenotype similar to that of CIPA (Yuan et al., 2013). Furthermore, HSAN type IID, caused by a mutation in *SCN9A*, was recently reported to show anhidrosis and cognitive impairment in addition to insensitivity to pain (Romagnuolo et al., 2024). Considering the heterogeneity of genes and phenotypes in HSAN, including CIPA, future investigations should be continued. Although insensitivity to pain, anhidrosis, and mental retardation are the typical findings of CIPA, heterogeneity exists in terms of phenotype and disease severity (Indo, 1993). Several variants are reported to show attenuated clinical manifestations in patients with CIPA (Jung et al., 2013; Ohto et al., 2004; Tanaka et al., 2012). A patient with compound heterozygote variants of p.Gly513Arg and p.Pro762Leu did not show severe mental retardation or self‐mutilation (Ohto et al., 2004). Furthermore, patients with p.Pro768Leu reportedly exhibit partially preserved pain sensation and normal intelligence (Jung et al., 2013; Tanaka et al., 2012). An in silico investigation into the phenotypic heterogeneity of CIPA patients revealed that p.Leu213Pro, Arg760Trp, and p.Pro767Leu were linked to normal intelligence. The structural similarities between proline and leucine, both having nonpolar side chains, contribute to the attenuated clinical symptoms (Liu et al., 2018). Another in vitro study conducted to elucidate the phenotype–genotype relationship investigated the effect of three *NTRK1* variants on protein structures and cellular functions and identified that the p.Cys300Ter variant was associated with normal intelligence. Misfolded conformers generated from p.Cys300Ter are not highly neurotoxic, and they can be promptly eliminated via autophagy (Miranda, Di Virgilio, et al., 2002).

In our study, four individual variants were identified: c.851‐33T>A (p.?), c.2020G>T (p.Asp674Tyr), c.2303C>T (p.Pro768Leu), and c.574‐156_850+1113del (exons 5‐7 del). The variant c.851‐33T>A (p.?) is the second most common variant found in Japanese CIPA patients and is regarded as the founder variant in East Asian populations (Lee et al., 2009; Miura et al., 2000; Wang et al., 2018). It causes aberrant splicing of intron 7 and the insertion of a 137‐bp section, resulting in the positioning of a premature stop codon at the 319th position (Liu et al., 2018; Miura et al., 2000). Proteins translated from these frameshift variants lack transmembrane domains and intracellular domains, including juxtamembrane region and the tyrosine kinase domain (Liu et al., 2018). The variant c.2020G>T (p.Asp674Tyr) is located in the tyrosine kinase domain and reduces the activity of the *NTRK1* receptor, instead of inactivating it. Adequate neural development has been observed in individuals homozygous for this variant, and a full inactivation of the corresponding allele is necessary for the disease‐associated expression pattern (Miranda, Di Virgilio, et al., 2002). The frequency of this variant is extremely low in the gnomAD v2.1.1 dataset (total allele frequency: 0.0000041, PM2). It has been observed as a compound heterozygote with other variants, suggesting the attenuated nature of this variant (López‐Cortés et al., 2020; Miranda, Di Virgilio, et al., 2002; Miura et al., 2000). The alternative variant situated in the tyrosine kinase domain, c.2303C>T (p.Pro768Leu), was documented to exhibit milder symptoms with retained cognitive function and pain sensation (Jung et al., 2013; Ohto et al., 2004; Tanaka et al., 2012). While preserved cognitive function was common in three cases, a difference in the degree of pain sensation was noted. Patients reported by Tanaka et al and Jung et al. showed partial pain sensation, whereas patients reported by Ohto et al showed pain insensitivity (Jung et al., 2013; Ohto et al., 2004; Tanaka et al., 2012).

The c.574‐156_850+1113del variant is a novel gross deletion variant that includes exons 5–7 (range of g.7071_11990 of NG_007493.1) and is situated in the extracellular domain. A comparable large deletion of g.6995_11999del was previously documented and was anticipated to lead to premature termination of translation, potentially causing the synthesis of an absent or truncated protein (Li et al., 2021). Compound heterozygotes with g.6995_11999del and g.1‐1258_10169del exhibit normal intelligence and no self‐mutilation, whereas compound heterozygotes with c.851‐33T>A (p.?) are associated with mental retardation and self‐mutilation (Li et al., 2021). As c.574‐156_850+1113del (exons 5‐7 del) is located near g.6995‐11999del, we expected that c.574‐156_850+1113del (exons 5‐7 del) would have a similar effect on the structure and function of *NTRK1*. However, it remains uncertain whether this extensive gross deletion is associated with an attenuated phenotype.

In our results, patients 1 and 2 showed pain insensitivity, global developmental delay, and self‐mutilation, whereas patient 3 showed relatively preserved pain sensitivity, normal development and intelligence, and no self‐mutilation. Furthermore, in addition to pain insensitivity, anhidrosis, mental retardation, and self‐mutilation, patient 1 showed severe musculoskeletal disorders and infectious diseases, which were not observed in patient 2. Patient 1 had homozygous c.851‐33T>A (p.?) that lacked a tyrosine kinase domain (Liu et al., 2018), whereas patient 2 showed compound heterozygous c.851‐33T>A (p.?) and c.2020G>T (p.Asp674Tyr), which were located in the tyrosine kinase domain and was reported to reduce the expression of the *NTRK1* receptor but not enough to inactivate it (Miranda, Di Virgilio, et al., 2002). Patient 3 had heterozygous c.2303C>T (p.Pro768Leu), which showed attenuated clinical manifestations (Jung et al., 2013), and a novel gross deletion c.574‐156_850+1113del (exons 5‐7 del). As CIPA is inherited in an autosomal recessive manner, it seems that patient 2 and patient 3 showed attenuated clinical manifestations because they had one “attenuated” variant, whereas patient 1 had homozygous c.851‐33T>A (p.?).

So far, there is no available treatment for CIPA. For patients or their families, preimplantation genetic analysis is the only option to prevent the birth of an affected child. Despite its importance, there is a lack of data regarding the prenatal assessment of CIPA patients. This study, unfortunately, did not reveal specific findings in prenatal screening, and no cases of preimplantation genetic analysis were identified.

## 5 CONCLUSIONS

We described the clinical and genetic features of three Korean CIPA patients. We identified one novel gross deletion variant and varying degrees of disease severity. Patients with variants previously reported to have an attenuated nature showed alleviated clinical manifestations. Our study expanded the spectrum of the *NTRK1* variants and provided additional information to further understand the genotype and phenotype relationship in CIPA. More information on the relationship between genotype–phenotype could be the key to elucidating the pathogenesis and clinical prognosis of CIPA.

## AUTHOR CONTRIBUTIONS

Jun Hee Cho and Beom Hee Lee designed this study. Jun Hee Cho, Beom Hee Lee, Yoon Hae Kwak and Mi‐Sun Yun collected data. June‐Young Koh, Young Seok Ju and Go Hun Seo conducted genetic sequencing. Jun Hee Cho and Beom Hee Lee interpreted the acquired data. Jun Hee Cho, Soojin Hwang, June‐Young Koh, Go Hun Seo and Beom Hee Lee drafted the manuscript, and Yoon Hae Kwak, Mi‐Sun Yum, June‐Young Koh, Young Seok Ju, Go Hun Seo, Ji‐Hee Yoon, Minji Kang, Hyo‐Sang Do, Soyoung Kim, Gu‐Hwan Kim and Hyunwoo Bae revised the manuscript. All authors read and approved the final manuscript. Beom Hee Lee is the corresponding author of this manuscript.

## FUNDING INFORMATION

This study was supported in part by the Bio and Medical Technology Development Program of the National Research Foundation (NRF) funded by the Korean Government (grant number: NRF‐2022R1A2C2091689), the Asan Institute for Life Sciences (Seoul, Republic of Korea) (2021IP0039), 3billions, inc. (Seoul, Republic of Korea), and Genome insight, inc. (San Diego, CA, USA) .

## CONFLICT OF INTEREST STATEMENT

The authors declare that there are no conflicts of interest.

## ETHICS APPROVAL AND CONSENT TO PARTICIPATE

This study was approved by the Institutional Review Board of Asan Medical Center, Seoul, Korea (IRB number: 2023‐1562) Written informed consent was obtained from all individuals. Parental consent was obtained from participants under 18 years old. This study complied with the Declaration of Helsinki.

## Data Availability

The datasets used during the current study available from the corresponding author on reasonable request.
